# Mechanism Analysis of UCP2 During the Oxidative Stress Injury of Intestinal Porcine Epithelial Cell Line-J2

**DOI:** 10.3390/ani15111654

**Published:** 2025-06-04

**Authors:** Weide Su, Chuanhui Xu, Hongping Jiang, Wenjing Song, Pingwen Xiong, Jiang Chen, Gaoxiang Ai, Qiongli Song, Zhiheng Zou, Qipeng Wei, Xiaolian Chen

**Affiliations:** 1 Institute of Animal Husbandry and Veterinary Medicine, Jiangxi Academy of Agricultural Sciences, Nanchang 330200, China; 2 Jiangxi Province Key Laboratory of Animal Green and Healthy Breeding, Nanchang 330200, China

**Keywords:** UCP2, oxidative stress, porcine intestinal epithelial cells, genipin, apoptosis

## Abstract

Oxidative stress poses a significant challenge in livestock production, impairing intestinal function, nutrient absorption, and overall animal performance. Uncoupling protein 2 (UCP2) is a mitochondrial regulator known for its protective effects against oxidative damage, but its specific function in porcine intestinal epithelial cells and its regulation by genipin—a natural UCP2 inhibitor with potential therapeutic properties—remains unclear. In this study, we cloned and overexpressed the porcine UCP2 gene in intestinal porcine epithelial cells (IPEC-J2), generating a stable UCP2-overexpressing cell line (IPEC-J2-UCP2). Under hydrogen peroxide-induced oxidative stress, UCP2 overexpression significantly improved cell viability, reduced reactive oxygen species (ROS) levels, and enhanced antioxidant enzyme activities (SOD, GPx, and CAT). Additionally, UCP2 upregulated the anti-apoptotic gene Bcl-2 and downregulated pro-apoptotic genes (Fas, Caspase-3, and Bax), indicating a protective role against oxidative stress-induced apoptosis. We also investigated the regulatory effects of genipin on UCP2. Under non-stress conditions, genipin mildly promoted anti-apoptotic gene expression. However, under oxidative stress, genipin strongly inhibited UCP2 expression, exacerbated ROS accumulation, reduced cell viability, and increased expression of pro-apoptotic markers, particularly Caspase-3 and Bax. These findings reveal that UCP2 plays a critical role in protecting porcine intestinal epithelial cells from oxidative injury and that genipin exerts context-dependent effects on cell fate by modulating UCP2. This study provides a mechanistic basis for targeting UCP2 to manage oxidative stress and improve intestinal health and performance in pigs.

## Simple Summary

Modern pig farming often faces challenges caused by oxidative stress, which happens when harmful molecules called reactive oxygen species build up in the body. This stress can damage the gut, reduce nutrient absorption, and harm overall pig health. In this study, we explored how a natural protein found in cells, called uncoupling protein 2, helps protect pig intestinal cells from this damage. We increased the levels of this protein in pig gut cells grown in the lab and found that the cells were better able to survive oxidative stress. They produced fewer harmful molecules and were less likely to activate genes that cause cell death. We also tested genipin, a plant-derived compound that can reduce the levels of this protective protein. We discovered that while genipin had little effect under normal conditions, it worsened the damage when the cells were under oxidative stress. These results show that uncoupling protein 2 plays an important role in defending pig gut cells from stress and that targeting this protein may help develop new strategies to improve gut health, support growth, and increase disease resistance in pigs—contributing to more sustainable and healthy livestock farming.

## 1. Introduction

Oxidative stress, characterized by an imbalance between ROS (reactive oxygen species) generation and antioxidant defense, is a key factor in the pathophysiology of many diseases, including inflammatory bowel disease and intestinal barrier dysfunction [1]. Excessive accumulation of ROS not only damages cellular components, such as lipids, proteins, and DNA, but also triggers apoptosis, ultimately compromising the integrity of the intestinal epithelium [2,3]. In livestock, including pigs, oxidative stress negatively affects intestinal health, leading to reduced nutrient absorption, increased intestinal susceptibility, and impaired growth performance [4,5]. Therefore, understanding the molecular mechanisms underlying the oxidative stress response of porcine intestinal cells is essential for developing strategies to improve animal health and productivity.

Among these molecular players, uncoupling protein 2 (UCP2) has attracted much attention due to its key role in regulating mitochondrial ROS generation and energy metabolism [6]. UCP2 is a member of the mitochondrial carrier protein family that is conserved across species and plays a central role in mitigating oxidative stress [7,8]. By uncoupling mitochondrial oxidative phosphorylation, UCP2 reduces ROS generation and enhances cellular antioxidant defense [9,10,11]. Studies in various species, including rodents and humans, have shown that UCP2 overexpression protects against oxidative damage and regulates apoptosis [12]. However, the specific role of UCP2 in porcine intestinal epithelial cells remains underexplored. Given the physiological and economic importance of gut health to pigs, investigating the functional role of UCP2 in porcine cells may not only provide biological insights but also offer practical applications for livestock management.

Genipin, a natural compound extracted from Gardenia jasminoides, has emerged as a potential modulator of UCP2 [13,14]. Genipin is considered a specific inhibitor of UCP2, and its inhibition has been shown to alter mitochondrial function, ROS production, and apoptosis in various cell types [15]. However, the effects of genipin on UCP2 activity and cellular responses to oxidative stress are complex and may vary across species, cell types, and environmental conditions [16,17,18]. In pigs, where oxidative stress is a common challenge in intensive farming environments [19,20], the interaction between UCP2 and genipin may have a significant impact on gut health and overall resilience to environmental stressors. Studying how genipin regulates UCP2 activity in porcine enterocytes could provide valuable insights into the management of oxidative stress in livestock.

To further address these gaps, this study aimed to explore the functional role of UCP2 overexpression and genipin intervention in porcine IPEC-J2 cells under oxidative stress conditions. First, we cloned the porcine UCP2 gene and established a stable UCP2-overexpressing cell line, IPEC-J2-UCP2, to investigate its adaptation to oxidative stress, focusing on cell viability, ROS levels, antioxidant enzyme activities, and apoptosis-related gene expression. Subsequently, we investigated the effects of genipin under normal and oxidative stress conditions, evaluating its effects on cell proliferation, UCP2 expression, and apoptosis regulation. By integrating molecular and functional analyses, this study provides important insights into the interaction between UCP2 and genipin in porcine enterocytes and its potential impact on improving porcine intestinal health and managing oxidative stress.

## 2. Materials and Methods

### 2.1. Construction of Lentiviral Vector Expressing UCP2 Gene

Total RNA was extracted from porcine jejunal tissue using an RNA extraction kit (Vazyme Biotech, RC113-01, Nanjing, China). Reverse transcription was performed using a reverse transcription kit (Thermo Fisher, Waltham, MA, USA) to synthesize cDNA. The coding sequence of the porcine UCP2 gene was retrieved from the GenBank database, and primers were designed to amplify the target sequence. The forward primer (ATAGAATTCGCCACCATGGTTGGATTCAAGGCCA) contained an EcoR I restriction enzyme site, and the reverse primer (ATAGGATCCAAAGGGAGCCTCCCGGGA) contained a BamH I restriction enzyme site. The PCR amplification reaction system was 50 μL, and the reaction conditions were as follows: 95 °C pre-denaturation for 5 min, 95 °C denaturation for 30 s, 58 °C annealing for 30 s, 72 °C extension for 1 min, 35 cycles, and 72 °C extension for 10 min. The PCR product was identified via 1.5% agarose gel electrophoresis. The DNA fragment was purified using a gel recovery kit (Biotechnology, Shanghai, China). The purified UCP2 gene fragment was connected to the lentiviral vector pHBLV-CMVIE-ZsGreen-Puro. The ligation product was transformed into DH5-α competent cells via heat shock at 42 °C for 45 s, and then revived by shaking in Luria–Bertani (LB) medium at 37 °C for 1 h. The transformation product was spread on LB agar plates containing ampicillin and incubated at 37 °C overnight. Positive colonies were screened using colony PCR. The PCR-positive clones were extracted using a plasmid extraction kit (TIANGEN Biotech, Beijing, China) and sent to Sangon Biotech (Shanghai) Co., Ltd. for sequencing to confirm that the UCP2 gene was correctly inserted into the lentiviral vector.

### 2.2. Production of Lentiviral Particles Carrying the UCP2 Gene

Lentiviral packaging was performed using a three-plasmid system consisting of pHBLV-CMVIE-ZsGreen-Puro-UCP2 (transfer plasmid), pMD2.G (envelope plasmid), and pSPAX2 (packaging plasmid). The plasmids were co-transfected into 293T cells using LipoFiter™ liposomal transfection reagent (Thermo Fisher, USA). The supernatant containing lentiviral particles was collected, filtered through a 0.45 μm filter to remove cell debris, and stored at −80 °C.

### 2.3. Establishment of UCP2-Overexpressing Porcine Intestinal Epithelial Cell Line (IPEC-J2-UCP2)

IPEC-J2 cells were infected with previously generated UCP2 lentivirus at a multiplicity of infection (MOI) of 10. After 48 h of infection, the medium was replaced with fresh medium containing 1 μg/mL puromycin to selectively enrich for successfully transduced cells. The cell line that stably grew after screening was named IPEC-J2-UCP2 and cryopreserved. As a vector control, IPEC-J2 cells were also transduced with an empty lentiviral vector expressing GFP alone (referred to as IPEC-J2-GFP). To confirm the overexpression of the UCP2 gene, total RNA was extracted from IPEC-J2-UCP2 cells, and the expression level of UCP2 mRNA was quantified using real-time quantitative PCR (RT-qPCR) using β-actin as the internal control.

### 2.4. Cell Viability Assay of IPEC-J2-UCP2 Cells Under Hydrogen Peroxide–Induced Oxidative Stress

The viability of IPEC-J2-UCP2 cells under oxidative stress was evaluated using the CCK-8 assay. Cells were seeded into 96-well flat-bottom plates at a density of 1 × 10^4^ cells/well in 100 μL of phenol red–free RPMI-1640 medium supplemented with 10% fetal bovine serum (FBS), 1% penicillin-streptomycin, and 2 mM L-glutamine. Oxidative stress was induced by adding 10 μL of 1 mM hydrogen peroxide (H_2_O_2_) to each well (final concentration: 91 μM) for 1 h. The control group was treated with an equal volume of PBS. After H_2_O_2_ treatment, cells were washed three times with PBS to remove residual H_2_O_2_. Subsequently, 100 μL of phenol red-free 1640 medium and 10 μL of CCK-8 reagent (Abcam, Cambridge, UK) were added and incubated at 37 °C for 2 h. The absorbance at 490 nm was measured using a microplate reader to determine cell viability. Viability percentage was calculated by normalizing the absorbance values of treated cells to those of untreated control cells, which were defined as 100% viability.

### 2.5. Measuring Intracellular Reactive Oxygen Species Levels Under Oxidative Stress Using Fluorescent Probes

Cells were stressed as described above. After stress treatment, cells were washed twice with PBS and incubated with specific fluorescent probes: DCFH-DA (10 μM) (Merck, Rahway, NJ, USA) for total free radical detection and DHE (5 μM) (Merck, USA) for superoxide anion radical measurement. The probes were added to the culture medium and incubated at 37 °C in the dark for 30 min. After incubation, cells were washed with PBS to remove excess probes and immediately detected using a microplate reader.

### 2.6. Evaluation of Antioxidant Enzyme Activities in IPEC-J2-UCP2 Cells Under Oxidative Stress

After oxidative stress treatment as described above, cells were lysed, and the supernatants were collected for enzyme activity analysis. Superoxide dismutase (SOD) activity was determined using the hydroxylamine method, glutathione peroxidase (GPx) activity was determined using a colorimetric method, and catalase (CAT) activity was assessed using the ammonium molybdate method. For specific methods, refer to the instructions provided by the respective test kits (JIANCHENG Bioengineering Institute, Nanjing, China). Absorbance values of each assay were recorded at the specified wavelength using a microplate reader. Total protein concentration was determined using a BCA protein assay kit (Beyotime, P0010, Shanghai, China), and enzyme activity was calculated based on the standard curve and normalized to the total protein concentration.

### 2.7. Apoptosis-Related Gene Expression Levels Under Oxidative Stress

Oxidative stress treatment was conducted as described above. Fluorescence quantitative PCR was used to analyze the expression levels of the anti-apoptotic gene Bcl-2 and the pro-apoptotic genes Fas, Caspase-3, and Bax. RNA was extracted from cells (~1 × 10^6^ cells) and reversely transcribed into cDNA. RT-qPCR was performed using specific primers (Table 1) for Bcl-2, Fas, Caspase-3, and Bax, respectively, with β-actin as an internal reference. The reaction system consisted of SYBR Green PCR Master Mix (QIAGEN, Hilden, Germany), the reaction volume was 20 μL, and the reaction conditions were as follows: 95 °C pre-denaturation for 5 min, 95 °C denaturation for 15 s, 60 °C annealing/extension for 30 s, and 40 cycles. The relative expression of the target gene was calculated using the 2^−ΔΔ^C_t_ method.

### 2.8. Proliferation of IPEC-J2 Cell Under Genipin Intervention

The proliferation of IPEC-J2 with or without genipin intervention under oxidative stress was measured using the MTT assay. Cells seeded into 96-well flat-bottom plates at a density of 1 × 10^4^ cells/well in 100 μL of phenol red-free RPMI-1640 medium supplemented with 10% fetal bovine serum (FBS), 1% penicillin-streptomycin, and 2 mM L-glutamine were treated with the following groups: genipin (100 μL of culture medium containing 50 μmol/L genipin), hydrogen peroxide (100 μL of culture medium containing 1 mmol/L H_2_O_2_), and hydrogen peroxide + genipin combination (100 μL of culture medium containing 1 mmol/L H_2_O_2_ and 50 μmol/L genipin). Cells were treated with 50 μmol/L genipin, a concentration selected based on previous studies demonstrating its efficacy in modulating mitochondrial function and inducing apoptosis without significant cytotoxicity [21]. To induce oxidative stress, cells were exposed to 1 mM H_2_O_2_ for 1 h, a condition commonly used in in vitro studies to mimic oxidative stress and induce apoptosis [22,23]. After 1 h of exposure to 1 mM H_2_O_2_ (with or without 50 μmol/L genipin), the treatment medium was aspirated and replaced with fresh medium (containing genipin or control medium as appropriate), and the cells were incubated for an additional 24 h at 37 °C in a 5% CO_2_ incubator. After the treatment, 10 μL of MTT solution (5 mg/mL) (Promega, Madison, WI, USA) was added to each well, and the culture was continued for 4 h. Then 150 μL of DMSO (Beyotime, China) was added to each well to dissolve the produced formazan crystals, and the absorbance at 490 nm was measured using a microplate reader.

### 2.9. Data Analysis

All experiments were performed with three independent biological replicates unless otherwise stated, and data are presented as mean ± SD and statistical analyses were conducted using an independent samples *t*-test for two-group comparisons using GraphPad Prism 10.2.1 (GraphPad Software Inc., San Diego, CA, USA). Significant values were declared if *p* < 0.05 (*), *p* < 0.01 (**), *p* < 0.001 (***), *p* < 0.0001 (****), and *p* ≥ 0.05 (ns).

## 3. Results

### 3.1. Generation and Validation of UCP2-Overexpressing IPEC-J2 Cell Line

**Figure 1:**
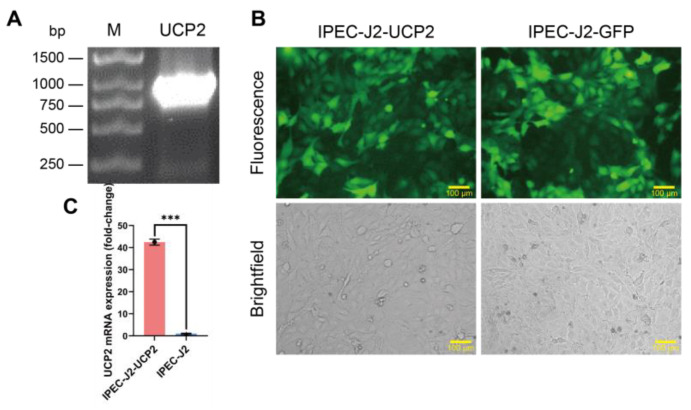
Construction and characterization of IPEC-J2-UCP2 cells. (**A**) Agarose gel electrophoresis of the PCR-amplified porcine UCP2 gene. Lane M: DNA ladder; Lane UCP2: PCR product of UCP2 gene (930 bp). (**B**) Fluorescence microscopy image of IPEC-J2-UCP2 and IPEC-J2-GFP cells. Both cell types exhibit GFP fluorescence due to transduction with GFP-expressing lentiviral vectors. IPEC-J2-UCP2 carries the UCP2 gene, while IPEC-J2-GFP serves as the vector control without the UCP2 insert. Scale bar: 100 μm. (**C**) Relative expression level of UCP2 mRNA in IPEC-J2-UCP2 and IPEC-J2 cells. Data are expressed as mean ± standard deviation (*n* = 3). Statistical significance was determined using an independent samples *t*-test: *p* < 0.001 (***).

The UCP2 gene was successfully amplified from porcine tissues. Agarose gel electrophoresis results showed that a DNA band of the expected size was observed at approximately 930 bp (Figure 1A). The UCP2 gene fragment obtained via PCR amplification was successfully integrated into the lentiviral vector and packaged into lentiviral particles using a three-plasmid system for transduction of IPEC-J2 cells. The cells were cultured in a medium containing 1 μg/mL puromycin to select successfully transduced cells. After several passages under puromycin selection, a stable cell line was established, called IPEC-J2-UCP2. To confirm successful transduction and expression, green fluorescence was observed in IPEC-J2-UCP2 cells under a fluorescence microscope (Figure 1B), as the lentiviral vector carried a GFP reporter gene that is co-expressed with UCP2. Fluorescence microscopy confirmed successful transduction in both IPEC-J2-UCP2 and IPEC-J2-GFP cells, the latter serving as a vector control expressing GFP without UCP2. The expression level of UCP2 mRNA in the IPEC-J2-UCP2 cell line was evaluated using RT-qPCR, and results showed that UCP2 mRNA expression in IPEC-J2-UCP2 cells was significantly up-regulated, showing a 42.46-fold increase compared to normal IPEC-J2 cells (Figure 1C). These results indicate the successful transcriptional overexpression of UCP2 in IPEC-J2 cells and suggest that the IPEC-J2-UCP2 cell line may serve as a useful model for subsequent functional studies of UCP2, pending further validation at the protein and activity levels. Having successfully established and verified the UCP2-overexpressing IPEC-J2 cell line, we next investigated how UCP2 modulation influences cellular responses to oxidative stress, focusing on redox status, antioxidant defenses, and apoptotic gene expression.

### 3.2. UCP2 Overexpression Enhances Oxidative Stress Resistance and Modulates Apoptotic Pathways in IPEC-J2 Cells

**Figure 2:**
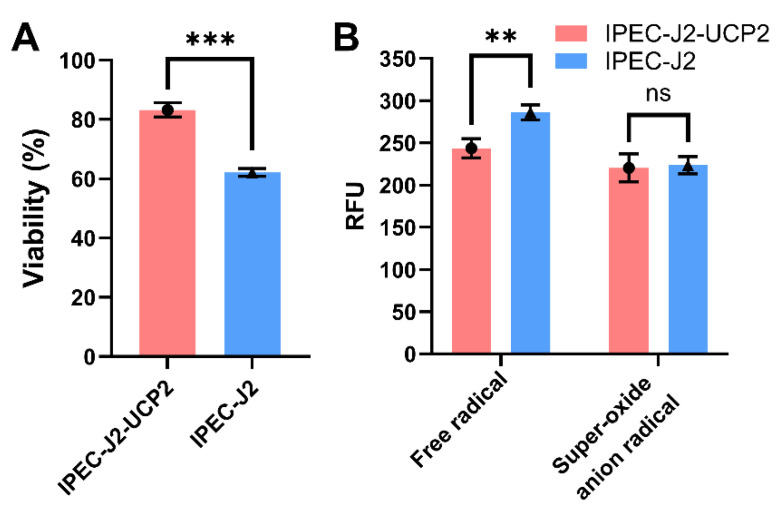
Cell viability (**A**) and ROS and superoxide anion radical levels (**B**) were measured in IPEC-J2 cells under hydrogen peroxide (H_2_O_2_)-induced oxidative stress. Cells were treated with 91 μM H_2_O_2_ for 1 h. Cell viability is expressed as a percentage relative to the untreated control group (set at 100%). Data are expressed as mean ± standard deviation (*n* = 3). Statistical significance was determined using an independent samples *t*-test: *p* < 0.01 (**) and *p* < 0.001 (***); ns indicates not significant.

Overexpression of UCP2 in IPEC-J2 cells significantly affected their ability to resist hydrogen peroxide (H_2_O_2_)–induced oxidative stress. Cell viability analysis showed that the viability of IPEC-J2-UCP2 cells under 91 μM H_2_O_2_ treatment was significantly higher than that of normal IPEC-J2 cells under oxidative stress conditions (*p* < 0.001), when viability was expressed relative to the untreated control (set as 100%), indicating enhanced cellular resistance to oxidative damage (Figure 2A). Further investigation of intracellular ROS levels revealed that the total ROS levels of IPEC-J2-UCP2 cells were significantly lower than those of normal cells (*p* < 0.01), suggesting an enhancement in overall antioxidant defense. However, no significant difference was observed in superoxide anion radical levels between the two groups (Figure 2B), possibly indicating that UCP2 preferentially mitigates specific types of ROS, such as hydrogen peroxide–derived radicals, rather than superoxide itself.

It is worth noting that hydrogen peroxide and superoxide anion differ in their intracellular generation mechanisms and targets. As only H_2_O_2_ was used in this study, further experiments using other oxidative stressors like menadione or rotenone will be necessary to comprehensively understand the spectrum of UCP2’s antioxidant effects.

**Figure 3:**
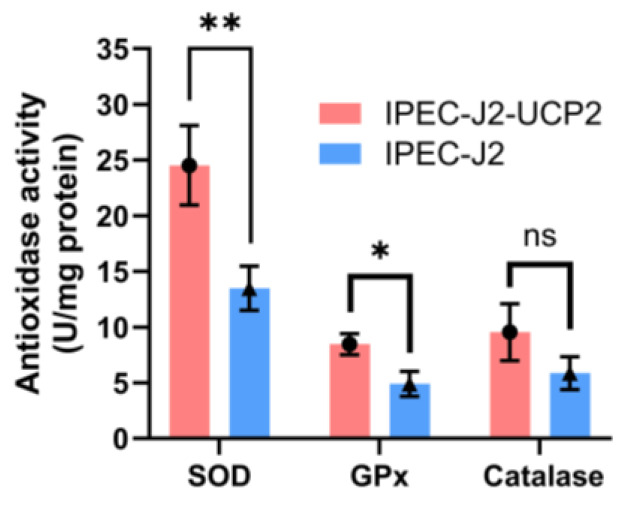
SOD, GPx, and CAT activities in cells were measured under hydrogen peroxide (H_2_O_2_)–induced oxidative stress. Data are expressed as mean ± standard deviation (*n* = 3). Statistical significance was determined using an independent samples *t*-test: *p* < 0.05 (*) and *p* < 0.01 (**); ns indicates not significant.

The activities of key antioxidant enzymes including SOD, GPx, and CAT in IPEC-J2-UCP2 cells continued to increase compared with controls (*p* < 0.05). These enhanced enzyme activities may help reduce total ROS levels and improve cell viability under oxidative stress (Figure 3).

**Figure 4:**
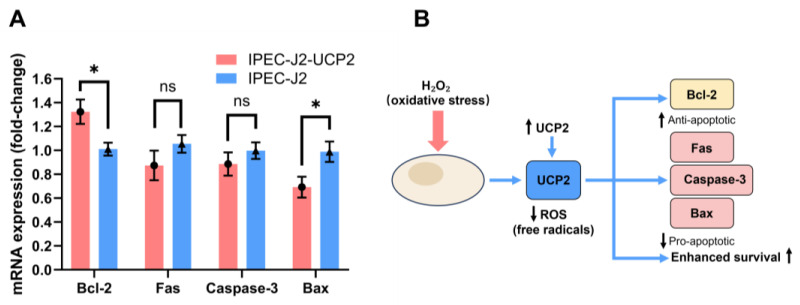
The expression levels of apoptosis-related genes in cells were measured under hydrogen peroxide (H_2_O_2_)–induced oxidative stress (**A**), and proposed mechanism through which UCP2 overexpression mitigates oxidative stress and apoptosis in IPEC-J2 cells. UCP2 reduces ROS accumulation under H_2_O_2_ stimulation, leading to decreased expression of pro-apoptotic genes (Fas, Caspase-3, and Bax) and increased expression of the anti-apoptotic gene Bcl-2, thereby enhancing cell survival (**B**). Data are expressed as mean ± standard deviation (*n* = 3). Statistical significance was determined using an independent samples *t*-test: *p* < 0.05 (*); ns indicates not significant.

In addition to affecting oxidative stress markers, UCP2 overexpression also modulated the expression of apoptosis-related genes. Under H_2_O_2_-induced stress, IPEC-J2-UCP2 cells exhibited a significant upregulation of the anti-apoptotic gene Bcl-2 compared to control cells (*p* < 0.05), whereas the expression of pro-apoptotic genes, including Fas, Caspase-3, and Bax, was suppressed, with Bax showing a statistically significant downregulation (*p* < 0.05) (Figure 4A). These results indicate that UCP2 not only alleviates oxidative stress but also confers protection against apoptosis. A schematic representation of this proposed mechanism is illustrated in Figure 4B, where UCP2 overexpression mitigates ROS accumulation and shifts the balance of apoptosis-regulating genes toward enhanced cell survival. Given the protective role of UCP2 under oxidative stress, we further explored the effects of its inhibition using genipin, a natural small-molecule UCP2 inhibitor, to understand the consequences of UCP2 suppression on redox homeostasis and apoptosis signaling.

### 3.3. Genipin Modulates Cell Proliferation, UCP2 Expression, and Apoptotic Pathways in IPEC-J2 Cells Under Oxidative Stress

**Figure 5:**
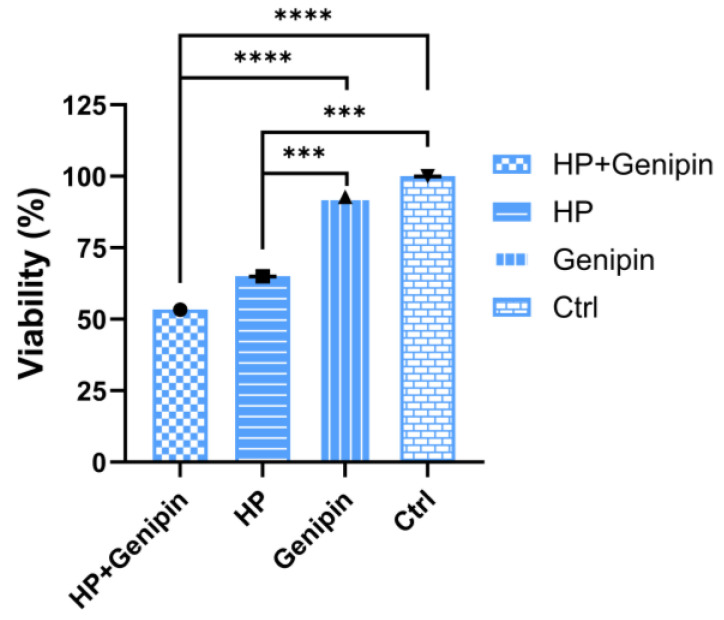
Cell viability of IPEC-J2 cells was measured under different treatments. Data are expressed as mean ± standard deviation (*n* = 3). Statistical significance was determined using an independent samples *t*-test: *p* < 0.001 (***) and *p* < 0.0001 (****). HP, hydrogen peroxide.

To further clarify the functional significance of UCP2 activity, we examined how pharmacological inhibition by genipin affects IPEC-J2 cell viability, oxidative stress responses, and apoptotic regulation. The effect of genipin intervention on IPEC-J2 cells under normal and oxidative stress conditions was analyzed, and it was found that there was a complex interaction between cell proliferation, UCP2 gene expression, and apoptosis regulation. In the absence of oxidative stress, 50 μmol/L genipin had no significant effect on the viability of IPEC-J2 cells compared to the control group as assessed via the MTT assay (Figure 5, Genipin vs. Ctrl). The same was true under conditions of oxidative stress (Figure 5, HP+Genipin vs. HP). However, when hydrogen peroxide induces oxidative stress, cell activity decreased significantly (Figure 5, HP vs. Ctrl, Genipin vs. HP, Genipin vs. HP+Genipin), and genipin further inhibited cell viability (Figure 5, HP+Genipin vs. Ctrl) (*p* < 0.0001), intensifying the inhibitory effect of oxidative damage on cell proliferation. This suggests that genipin may amplify the decrease in cell viability caused by oxidative stress by regulating cellular stress responses (Figure 5).

**Figure 6:**
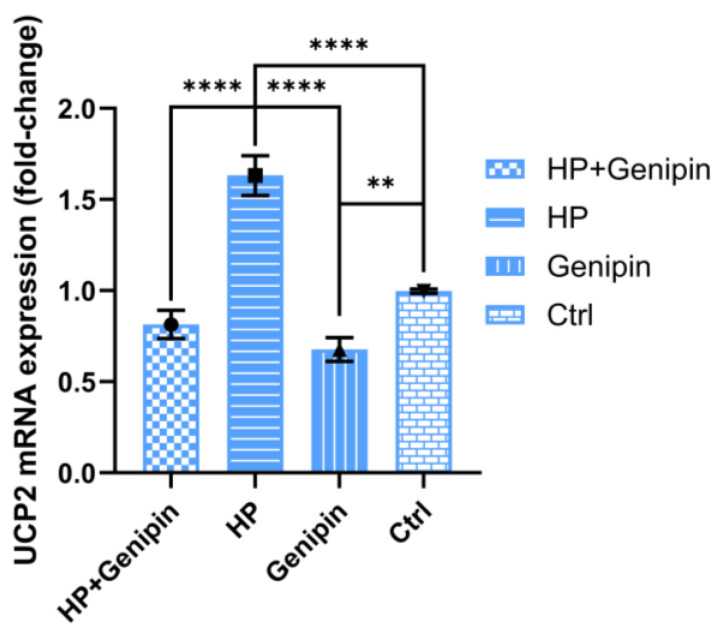
Relative expression levels of UCP2 mRNA of IPEC-J2 cells were measured under different treatments. Data are expressed as mean ± standard deviation (*n* = 3). Statistical significance was determined using an independent samples *t*-test: *p* < 0.01 (**) and *p* < 0.0001 (****). HP, hydrogen peroxide.

Consistent with its effect on cell proliferation, genipin significantly reduced UCP2 mRNA expression in IPEC-J2 cells. In the absence of stress, UCP2 mRNA levels were reduced by 31.95% compared with the control group (Figure 6, Genipin vs. Ctrl) (*p* < 0.01). This inhibition was more pronounced under oxidative stress, with UCP2 mRNA levels reduced by 50.07% compared with the hydrogen peroxide group (Figure 6, HP+Genipin vs. HP) (*p* < 0.0001). Oxidative stress significantly increased UCP2 mRNA levels (Figure 6, HP vs. Ctrl) (*p* < 0.0001), although the effect was not obvious during genipin intervention (Figure 6, HP+Genipin vs. Genipin), which reflected the distinct bidirectional regulation of oxidative stress and genipin (Figure 6, Genipin vs. HP) (*p* < 0.001). These results highlight the role of genipin in downregulating UCP2 (Figure 6, HP+Genipin vs. Ctrl), potentially contributing to its effects on cell proliferation under stress (Figure 6).

**Figure 7:**
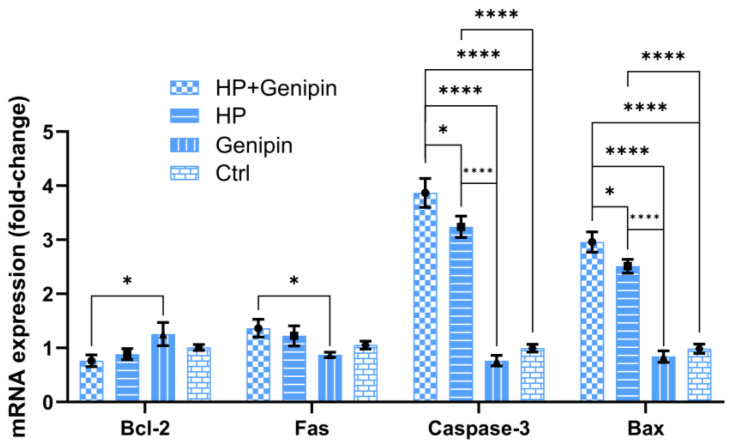
Expression levels of apoptosis-related genes (Bcl-2, Fas, Caspase-3, and Bax) of IPEC-J2 cells were measured under different treatments. Data are expressed as mean ± standard deviation (*n* = 3). Statistical significance was determined using an independent samples *t*-test: *p* < 0.05 (*) and *p* < 0.0001 (****). HP, hydrogen peroxide.

In addition to modulating UCP2 expression, genipin also significantly altered the expression of apoptosis-related genes. Under non-stress conditions, genipin enhanced the expression of the anti-apoptotic gene Bcl-2 (*p* > 0.05) while reducing the expression of pro-apoptotic genes, including Fas, Caspase-3, and Bax (Figure 7, Genipin vs. Ctrl) (*p* > 0.05). These effects suggest that genipin exerts a protective effect under basal conditions by shifting the apoptotic balance toward cell survival. However, under oxidative stress, genipin reversed this trend, which suppressed Bcl-2 expression and up-regulated pro-apoptotic genes, especially Caspase-3 and Bax (Figure 7, *p* < 0.05). Furthermore, the effect of oxidative stress on apoptosis-related genes in IPEC-J2 cells is complex. The regulation of oxidative stress on the anti-apoptotic gene Bcl-2 and the pro-apoptotic gene Fas was not significant; however, it strikingly up-regulated the apoptosis-promoting genes Caspase-3 and Bax (Figure 7, HP vs. Ctrl) (*p* < 0.0001). A similar trend was observed after the intervention of genipin (Figure 7, HP+Genipin vs. Ctrl), with a more severe impact on the pro-apoptotic genes Caspase-3 and Bax (Figure 7, HP vs. Genipin, HP+Genipin vs. Genipin). These findings imply that genipin may promote stress-induced apoptosis, further leading to reduced cell viability under oxidative stress (Figure 7).

## 4. Discussion

The results of this study provide valuable insights into the effects of UCP2 overexpression and genipin intervention on porcine intestinal epithelial cells under oxidative stress. Data highlight the dual role of UCP2 in promoting cellular resilience to oxidative damage and regulating apoptosis, as well as the complex regulatory role of genipin, particularly under conditions of oxidative stress. Oxidative stress is a key challenge in modern intensive farming systems, so these results have important implications not only for basic biological understanding but also for livestock health management, where oxidative stress significantly affects production and animal welfare.

Under hydrogen peroxide–induced oxidative stress, UCP2 overexpression significantly enhanced cell viability, reduced total ROS levels, and increased antioxidant enzyme activities (SOD, GPx, and CAT), demonstrating its protection against oxidative damage. These results are consistent with previous studies showing that UCP2 acts as a mitochondrial regulator to reduce oxidative stress by reducing mitochondrial ROS production and enhancing antioxidant defense [24,25,26]. These findings highlight the potential selectivity of UCP2 toward certain ROS species. The differential response to H_2_O_2_-induced stress underscores the need to explore UCP2’s function using various ROS inducers, such as menadione, to determine whether its antioxidant effects are ROS-type specific. Furthermore, up-regulation of the anti-apoptotic gene Bcl-2 and down-regulation of pro-apoptotic genes (Fas, Caspase-3, and Bax) were observed, further emphasizing the anti-apoptotic properties of UCP2, which may contribute to improved cell survival. Conditions such as heat stress, dietary imbalance, and infection often lead to increased ROS production in pigs, negatively impacting intestinal health and systemic homeostasis [27,28]. The observed protective effects of UCP2 in porcine intestinal epithelial cells suggest that enhancing UCP2 expression may be a feasible strategy to improve porcine intestinal resilience, thereby contributing to enhanced nutrient absorption, growth performance, and disease resistance.

Our study’s findings align with and expand upon emerging knowledge of porcine UCP2 in various tissues. For example, Zhou et al. (2022) demonstrated that in pig oocytes, the mitochondrial antioxidant Mito-Q downregulates UCP2 expression, lowers ROS levels, and enhances oocyte maturation [29]. Similarly, in porcine granulosa cells, antioxidant treatments increase activities of SOD, CAT, and GPx and shift apoptosis markers toward cell survival (upregulating Bcl-2 and suppressing Bax) [30], consistent with our observations under genipin treatment. These pig-specific studies suggest that modulation of UCP2 critically affects redox balance and apoptotic signaling in porcine cells. Cold-stress experiments in pigs further illustrate this context-dependence. Guo et al. (2024) noted that UCP2 expression in pig adipose under cold stress can change in either direction across studies [31], underscoring that UCP2’s role varies by tissue and environmental challenge. Together, these comparisons indicate that our results are not anomalous but fit a pattern where porcine UCP2 influences oxidative stress and cell survival. Mechanistically, our data highlight how UCP2 inhibition by genipin impacts the ROS–antioxidant–apoptosis axis. The ROS surge in our study induced compensatory antioxidant responses; activities (and in other systems, expression) of SOD, GPx, and CAT were elevated in treated tissues, reflecting an attempt to restore redox balance [32]. However, the excess ROS also activated apoptotic pathways. We found increased expression of pro-apoptotic genes (Bax, Fas, and caspase-3) and decreased anti-apoptotic Bcl-2. This pattern matches other systems where loss of UCP2 led to apoptosis; for instance, UCP2 blockade in stressed neurons increased caspase-3 and reduced antioxidant enzyme activities [33]. Likewise, the elevated UCP2 expression helps suppress ROS and maintains the Bcl-2/Bax balance [34], implying that without UCP2’s action, cells are driven toward apoptosis. In sum, our mechanistic interpretation is that genipin’s inhibition of UCP2 removes a key control on ROS, causing ROS accumulation that triggers antioxidant defense and shifts gene expression toward cell death.

Interestingly, under non-stress conditions, inhibition of UCP2 by genipin was associated with significant downregulation of pro-apoptotic genes Fas, Caspase-3, and Bax, without significantly affecting the expression of the anti-apoptotic gene Bcl-2. This selective gene regulation suggests that genipin may exert a cytoprotective effect upon UCP2 inhibition by shifting the balance toward cell survival through inhibition of pro-apoptotic pathways rather than directly enhancing anti-apoptotic signaling. This result is consistent with previous reports that have shown that UCP2 inhibition leads to enhanced mitochondrial ROS generation [35,36], which, at controlled levels, may activate adaptive survival pathways and enhance cellular resistance to apoptosis. In contrast, during oxidative stress, paradoxical trends in apoptotic genes were observed following inhibition of UCP2 expression by genipin, suggesting that cell survival or regulatory mechanisms may not only revolve around apoptosis inhibition but may also involve promoting damage-selective apoptosis to maintain overall cellular integrity when UCP2 is inhibited by genipin. In conclusion, the above findings suggest that inhibition of UCP2 gene expression by genipin orchestrates a complex balance between cell survival and apoptosis that is context-dependent and influenced by the presence or absence of oxidative stress.

A limitation of this study is that UCP2 overexpression was confirmed at the mRNA level but not at the protein level due to experimental constraints. Future studies including protein-level validation (e.g., Western blot) will help to further support the mechanistic interpretation of our findings. Additionally, our analysis was limited to four well-established apoptosis-related genes; future studies incorporating broader transcriptomic or pathway-targeted profiling (e.g., p53/PUMA axis) will help uncover additional regulatory mechanisms involved in UCP2 modulation.

From a broader perspective, these findings contribute to the understanding of the physiological role of UCP2 in livestock species, laying the foundation for translational research aimed at improving animal health and productivity. Although genipin was effective in modulating UCP2 expression in vitro, its bioavailability and safety in pigs remain unclear. Future in vivo studies should evaluate the pharmacodynamics and tolerability of genipin or consider safer alternatives such as resveratrol or metformin as UCP2 modulators. Further in vivo studies are needed to confirm these effects and explore the feasibility of manipulating UCP2 activity in pigs through genetic or pharmacological approaches. Furthermore, the addition of dietary antioxidants or UCP2 modulators to livestock feed may be a new strategy to mitigate oxidative stress in intensive farming systems, thereby improving overall pig health and performance.

## 5. Conclusions

This study highlights the critical role of porcine UCP2 in protecting intestinal epithelial cells from oxidative damage induced by H_2_O_2_ while revealing environmentally relevant effects of genipin as a modulator of UCP2. These findings provide a basis for developing strategies to manage oxidative stress in pigs and may have applications in enhancing intestinal health, disease resistance, and overall livestock productivity.

## Figures and Tables

**Table 1:** Primer sequences used for RT-qPCR.

Product (bp)	Sequence (5′–3′)	GenBank Accession No.	Gene Name
181	F: GGACCTGACCGACTACCTCAT	DQ452569	β-actin
	R: GGGCAGCTCGTAGCTCTTCT		
180	F: ACCTGAATGACCACCTAGAGC	NM_214285	Bcl-2
	R: TCCGACTGAAGAGCGAAC		
103	F: TGATGCCCAAGTGACTGACC	NM_213839	Fas
	R: GCAGAATTGACCCTCACGAT		
190	F: GTGGGACTGAAGATGACA	NM_214131	Caspase-3
	R: ACCCGAGTAAGAATGTG		
154	F: CCGAAATGTTTGCTGACG	XM_003127290	Bax
	R: AGCCGATCTCGAAGGAAGT		

## Data Availability

All the data generated during the current study are included in this manuscript.
